# The LITTability study - evaluation of the applicability of LITT in a real-world cohort of glioma patients

**DOI:** 10.1007/s10143-025-03644-5

**Published:** 2025-06-03

**Authors:** Manuel Kaes, Vincenzo Rondinelli, Sandro M. Krieg, Martin Jakobs

**Affiliations:** 1 Department of Neurosurgery, Heidelberg University Hospital, Im Neuenheimer Feld 400, 69120 Heidelberg, Germany; 2 Division for Stereotactic Neurosurgery, Department of Neurosurgery, Heidelberg University Hospital, Im Neuenheimer Feld 400, 69120 Heidelberg, Germany; 3 Medical Faculty, Heidelberg University, Grabengasse 1, 69117 Heidelberg, Germany

**Keywords:** LITT, Laser interstitial thermal therapy, Glioma treatment, Stereotactic laser therapy, Applicability of LITT

## Abstract

Laser-interstitial thermal therapy (LITT) is a minimally invasive technique used in neurosurgery for ablation of epileptic foci and malignant lesions, especially for glioma located in regions that pose high surgical risk. Current research mainly focuses on maximizing the safety of the procedure and proving the non-inferiority compared to open resection of glioma. However, data regarding the current applicability in real-time cohorts are lacking. The goal of this study is to evaluate the real-world applicability of LITT in glioma patients, specifically focusing on those who had undergone stereotactic biopsy, and to define limiting factors. For this retrospective study, we analyzed *n* = 207 glioma patients from a monocentric stereotactic surgery database over a 5-year period (2018–2022). Clinical, histopathological and radiological data were assessed. To define a lesion suitable for LITT, a two-step approach was used. In a first step, predefined selection criteria were applied consisting of a Karnofsky Performance Score of 70 or higher, an ASA Score of 3 or less, MRI compatibility, and glioma presenting as a single or bifocal lesion. In a second step, the LITT simulation was performed with additional criteria consisting of at least 90% possible ablation volume, a safe trajectory with avoidance of vessels, and optimal lesion accessibility without brainstem involvement. Out of 207 patients, 137 cases met initial preselection criteria, while 36 cases (17.4%) were ultimately deemed suitable for LITT post-simulation. Common exclusion factors included multifocal lesions, irregular lesion shape, and size constraints. Among suitable cases, 94.4% had unifocal lesions. For 44.4% of cases, only a single catheter was needed, with the number of ablation points varying from one to twelve per trajectory. The average lesion diameter for LITT-suitable cases was 26.4 mm. Even though LITT offers a promising alternative for glioma not suitable for open resection, the current application is limited. Main reasons were due to lesion morphology and size. Enhancing LITT applicability could involve addressing constraints posed by lesion geometry and volume. Prospective studies comparing LITT with conventional resection could better define the subset of glioma patients who may benefit most, advancing the potential for LITT in clinical neurosurgical practice.

## Clinical trial number

Not applicable.

## Introduction

Laser-interstitial thermal therapy (LITT) has emerged in neurosurgery over recent years. This technique is based on the application of nonionizing radiation (light) to tissue, in which the energy is converted into thermal energy (heat) causing cellular necrosis and thermal damage to the surrounding tissue [1, 2]. Due to its minimally invasive nature, facilitated by stereotactic guidance and real-time monitoring of heat distribution via intraoperative magnetic resonance imaging (MRI), LITT is already considered an alternative to traditional craniotomy for certain brain pathologies, such as radiation necrosis, metastases, primary brain tumors, meningiomas, and epileptic foci [2–4]. The benefits of LITT treatment include a lower risk of surgical complications compared to craniotomy [5]. It is also reported that the hyperthermia application disrupts the brain-blood barrier which could enhance delivery of chemotherapy to brain tissue [6]. Furthermore, synergistic effects with a better effect of a postsurgical radiation therapy are reported [7]. As a result, LITT is considered a promising treatment option for non-resectable primary brain tumors. In glioma treatment, the extent of resection (EOR) is one of the main factors influencing overall survival (OS) [8, 9]. For tumors located in deep or eloquent brain regions this goal often cannot be achieved due to the high risk of neurological deficits [10]. In such cases, stereotactic biopsy followed by radio-chemotherapy is considered the standard of care [7]. Recent studies have shown that additional LITT can be performed without increasing complication rates and thereby offering improved OS and progression-free survival (PFS) [11, 12]. However, whether LITT could be an alternative with similar outcome for glioma suitable for open resection still needs to be elucidated, although recent studies suggest a similar outcome [13]. Therefore, LITT offers a treatment option for tumors, where a safe open resection is not suitable and might also be an alternative for tumors which can be resected via open surgery in very selected cases. Especially in recurrent high grade glioma, where open surgical resection is associated with a higher risk of perioperative complications, LITT has emerged as a treatment option in real time cohorts for cytoreduction [7]. However, despite the promising results from recent studies there are several limiting factors for LITT application in real world cohorts. Successful outcomes are only observed when high ablation volumes (typically at least over 70%) are achieved, with the most beneficial effect over 90% [14–16]. The stereotactic approach confines LITT to small, regularly shaped lesions that are safe reachable over a straight trajectory. Also the clinical state of the patient is a limiting factor with a Karnofsky performance Score (KPS) less than 70% being an exclusion criterion for most of the recent LITT studies [14, 16]. Because general anesthesia is required, only patients suitable for general anesthesia, determined by their American Society of Anesthesiologist score (ASA), can be considered and recent data showed a positive correlation of the ASA Score and the 30 day mortality after stereotactic biopsies [17]. Furthermore, because LITT requires intraoperative MRI, only patients without non-MRI compatible implants (e.g. cardiac pacemakers) can undergo the procedure. The objective of this study was to evaluate the applicability of LITT in a real-world cohort of glioma patients who had undergone stereotactic biopsy at our institution, identifying those who could potentially be candidates for the LITT procedure.

## Methods

### Cohort

For this study, a retrospective database analysis of the stereotactic surgery database of the Division for Stereotactic Neurosurgery, Department of Neurosurgery of the Heidelberg University Hospital, from the years 2018 to 2022 was performed. Overall, 323 cases were screened. All patients with glioma as the final histopathological diagnosis were enrolled (*n* = 207). Clinical data regarding histopathological diagnosis, Karnofsky Performance Scale (KPS), ASA-Score, comorbidities and previous cranial surgeries were collected from the admission and discharge letters as well as the anesthesiologists reports and surgical reports. Radiological data were assessed by analyzing the radiological reports and pictures taken out of the Picture Archiving and Communication System (PACS). The maximal diameter of the lesion was measured in the plane of the largest extension of the lesion. In cases of contrast-enhancing lesions, the contrast enhancing areas were used, in non-enhancing lesions the Flair-Hyperintensity was used for measurement.

### LITT simulation

After exclusion of cases in the preselection process (see below) the LITT simulation was performed. LITT simulation was conducted by two neurosurgeons (one resident and one attending) using the inomed planning software (iPS 7.0, inomed Medizintechnik GmbH, Emmendingen, Germany). This software allows the simulation of predefined volumes and shapes alongside a chosen stereotactic trajectory. Several volumes can be placed along the trajectory to simulate the situation when the laser fiber is pulled back within the laser catheter to create elongated ablation volumes with multiple ablation points. The volumes for a single laser ablation were manually chosen for the given average lesion shape, diameter and position alongside the trajectory according to personal experience and in communication with the LITT system’s (Visualase™, Medtronic, Minneapolis, MN, USA) manufacturer. The standard ablation volumes can be placed along the stereotactic trajectory to simulate the position of the ablation. This also allows simulation of the combined ablation volume of multiple ablations created via a fiber pull-back inside the laser catheter if necessary to fully cover the desired lesion. The available laser fibers included two different laser diffusion sizes: a 3 mm and a 10 mm long laser diffusion tip fiber.

The software also allows for volumetry of the desired lesion to be ablated as well as the volumetry of the parts of the lesion outside of the ablation volume. To assess LITTability, the difference between target volume and volume outside the simulated lesion volume was calculated. In cases of contrast-enhancing lesions, the ablation volume was calculated based on the contrast-enhancing area, while in non-enhancing lesions, ablation volume was calculated based on the FLAIR-hyperintense area. The trajectories needed to avoid conflicts with vessels and sulci. The number of trajectories, the number of ablations along the trajectories, the maximal diameter as well as the need for an additional burr-hole was documented.

### Definition of inclusion and exclusion criteria

Inclusion and exclusion criteria for this study were selected based on a thorough literature review and aligned with the criteria used in the ongoing HI-SMILE study at our institution. LITTability was determined according to the following parameters (see also Table 1):

Preselection Criteria:

1. Histopathological diagnosis: Only patients with any type of confirmed glioma were included; all non-glioma cases were excluded.
2. Lesion characteristics: Only uni- or bifocal lesions were included; multifocal lesions were excluded.
3. Karnofsky Performance Score (KPS): Patients with a KPS of 70 or higher were included, while those with a KPS below 70 were excluded.
4. American Society of Anesthesiologists (ASA) Score: Patients with an ASA score of 3 or lower were included; those with an ASA score above 3 were excluded.
5. MRI compatibility: Patients with contraindications for MRI, such as MRI-incompatible pacemakers, cochlear implants, or severe obesity, were excluded.

LITT Simulation Criteria:

A lesion was considered to be suitable for LITT only if all of the following criteria were matched:

1. An ablation volume of 90% or more was achievable.
2. Ablation volume could be achieved with one or two trajectories.
3. The trajectories were safe without any involvement of eloquent areas or vascular, ventricular or sulcal conflicts. Whenever available fiber tracking for motor function was part of the considerations for trajectory choice.
4. The location of the lesion was not in the brainstem.

**Table 1:**
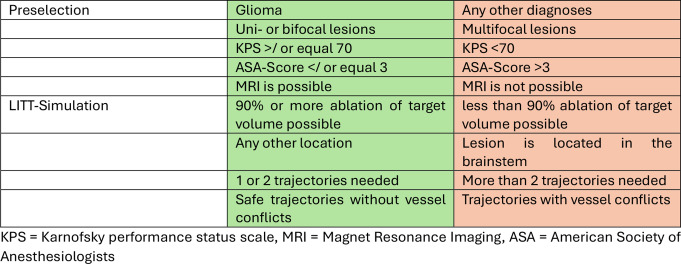
LITTability criteria

### Statistics

Nominal and ordinal variables are presented as numbers, frequencies, and medians; for continuous variables, the mean, range, and standard deviation are shown. All statistics were performed using SPSS Statistics (IBM, Version 29.0, Armonk, NY; USA).

## Results

### Cohort

A total of 207 patients with a histopathological diagnosis of glioma were enrolled in the study. Of these, 118 patients (57%) were male, and 89 patients (43%) were female. The mean age at the time of biopsy was 59.25 years (range: 8–89 years, SD 17.83). The most common tumor localizations were the corpus callosum (*n* = 42, 20.3%), frontal lobe (*n* = 37, 17.9%), temporal lobe (*n* = 34, 16.4%), and thalamus (*n* = 23, 11.1%). Most patients (*n* = 169, 81.6%) were diagnosed with WHO grade 4 glioma. Fourteen patients (6.8%) had recurrent tumors, while 193 patients (93.2%) had primary gliomas. Additionally, *n* = 167 gliomas (80.7%) were contrast-enhancing lesions while *n* = 40 gliomas (19.3%) were non-enhancing (FLAIR-hyperintense only). Other baseline characteristics (including IDH-mutation, MGMT-methylation, number of specimens, pre-surgical NIHSS, intraoperative visualizing and biopsy instruments) can be found in Table 2.

**Table 2:** Baseline characteristics

Parameter		*n*	%	Range, Mean, SD
All cases		207	100	
Sex	Male	118	57	
	Female	89	43	
Age				8–89, 59.25, 17.83
Pediatric patients		10	4.8	
Localization	Frontal	37	17.9	
	Parietal	14	6.8	
	Temporal	34	16.4	
	Occipital	5	2.4	
	Basal ganglia	18	8.7	
	Thalamus	23	11.1	
	Brainstem	13	6.3	
	Cerebellum	1	0.5	
	Insula	8	3.9	
	Corpus Callosum	42	20.3	
	Periventricular	7	3.4	
	Hypothalamic	4	1.9	
	Other	1	0.5	
WHO	1	7	3.4	
	2	18	8.7	
	3	7	3.4	
	4	169	81.6	
Recurrent tumor	Yes	14	6.8	
	No	193	93.2	
IDH-status	Mutated	31	15	
	Wildtype	176	85	
MGMT-status	Methylated	93	45	
	Wildtype	87	42	
	Not specified	27	13	
Intraoperative Visualizing	MRI	152	73.4	
	CT	55	26.6	
Contrast-enhancement	Yes	167	80.7	
	No	40	19.3	
Biopsy instrument	Forceps	199	96.1	
	Baklund Needle	8	3.9	
Number of specimens				3–31, 16.18, 4.967
NIHSS preoperative				0–20, 2.06, 2.798

WHO = World Health Organization, IDH = Isocitrate Dehydrogenase, MGMT = O6-methylguanine-DNA methyltransferase, NIHSS = National Institutes of Health Stroke Scale

### Preselection

**Fig. 1:**
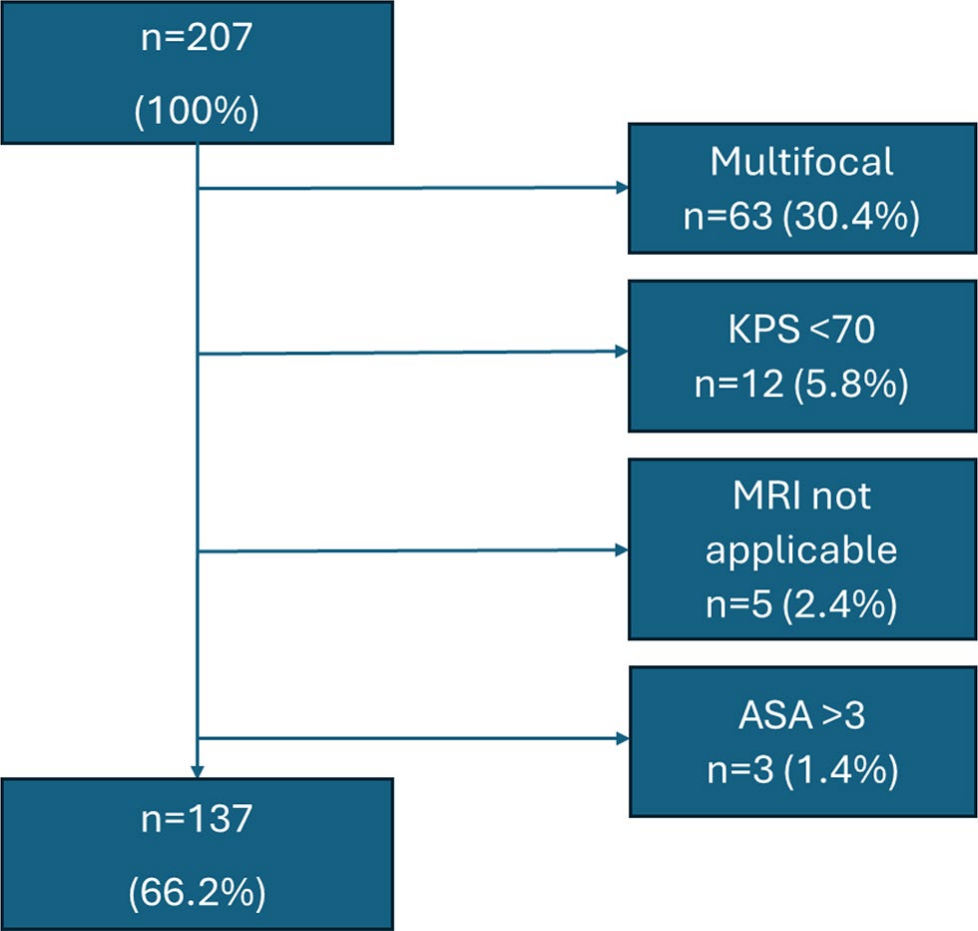
Flowchart of Preselection. KPS = Karnofsky performance status scale, MRI = Magnet Resonance Imaging, ASA = American Society of Anesthesiologists

In the first step, preselection criteria were applied to all 207 glioma cases. Multifocal lesions were present in 63 cases (30.4%). Twelve patients (5.8%) had a Karnofsky Performance Status (KPS) below 70. In five cases (2.4%), MRI was not possible due to severe obesity, the presence of a pacemaker, or a cochlear implant. Three patients (1.4%) had an ASA score greater than 3. All cases with the above-mentioned criteria were considered non-feasible for a LITT procedure and were therefore excluded from further analysis. It is important to note that some patients presented with more than one exclusion criterion. After preselection, the total number of patients eligible for LITT was reduced to 137 (66.2%). For the flowchart of the preselection process, see Fig. 1.

### LITT-Simulation

**Fig. 2:**
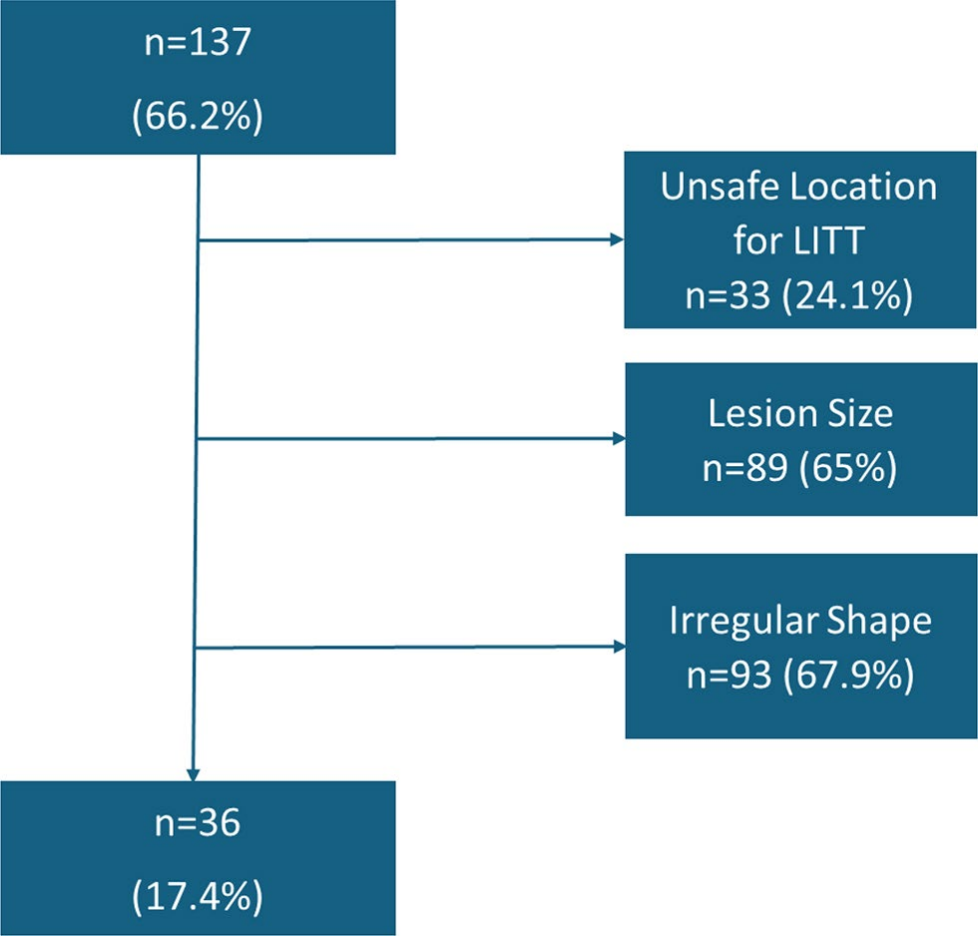
Flowchart for LITT-simulation

In the second step, all cases passing preselection were simulated for the LITT-procedure. The goal was to achieve at least 90% of ablation volume with one or two catheters without causing any vascular or sulcal conflicts as well as preventing damage to eloquent areas (e.g. brainstem). This goal was met in 36 patients (26.3%) out of the 137 simulated cases. In 33 cases (24.1%), the lesion was not safely accessible. In 89 cases (65%), the lesion volume was deemed too large for the LITT procedure. Additionally, the shape of the lesion was unsuitable for LITT in 93 cases (67.9%). It should be noted that more than one of the above-mentioned limiting factors was often present in a single case. Ultimately, the total number of gliomas considered suitable for LITT was 36 (17.4%). For further details, refer to Fig. 2, which outlines the flowchart of the LITT simulation process.

### LITTable glioma

Following the preselection and simulation process, 36 gliomas (17.4%) were deemed suitable for the LITT procedure. Of these, 34 (94.4%) were unifocal, and 2 (5.6%) were bifocal. In 16 cases (44.4%), 90% ablation volume was achieved using a single catheter, while in 20 cases (55.6%) a second catheter was required. A 3 mm fiber was used in 5 cases (13.9%), a 10 mm fiber in 23 cases (63.9%), and both fiber types in 8 cases (22.2%). The mean number of ablation points along the catheter(s) was 3.83 (SD 2.48, range 1–12). The mean maximal diameter of the suitable lesions was 26.42 mm (SD 8.7, range 8–54 mm). For further details regarding gliomas suitable for LITT, refer to Table 3.

**Table 3:** Characteristics of littable glioma

		*n*	%	Range, Mean, SD
LITTable glioma		36	100	
Unifocal		34	94.4	
Bifocal		2	5.6	
Number of catheters	1	16	44.4	
	2	20	55.6	
Catheter type	3 mm	5	13.9	
	10 mm	23	63.9	
	both	8	22.2	
Ablations along catheter	1	5	13.9	1–12, 3.83, 2.48
	2	10	27.8	
	3	4	11.1	
	4	4	11.1	
	5	4	11.1	
	6	4	11.1	
	7	3	8.3	
	8	1	2.8	
	12	1	2.8	
Maximal diameter in mm				8–54, 26.42, 8.7

### Assessment of LITTability

From 207 enrolled glioma, *n* = 36 (17.4%) were considered LITTable after preselection and LITT-Simulation. The majority of gliomas *n* = 171 (82.6%) were considered not suitable for LITT. Main reasons for Exclusion in the preselection process were multifocal appearance (*n* = 63, 30.4%) and a low KPI (*n* = 12, 5.8%). Main reasons for exclusion in the LITT-Simulation process were unsuitable shapes and sizes to achieve safe, 90% ablation (67.9% and 65%).

## Discussion

### Inclusion and exclusion criteria

The Karnofsky Performance Status (KPS) is widely utilized as a cut-off value in clinical oncology studies. In most neurosurgical research, a KPS of 70 is commonly accepted as the threshold for determining eligibility for surgical intervention. This cut-off is also typically applied in prospective studies on LITT [14, 16] The American Society of Anesthesiologists (ASA) classification has been recognized as a predictor of perioperative complications and mortality risk in previous case series involving stereotactic biopsies [17]. Specifically, an ASA score of 3 or higher has been correlated with an increased risk of mortality. In oncological patients, we believe that, based on a comprehensive risk-benefit assessment, surgical intervention under general anesthesia is appropriate for those with an ASA score of ≤ 3. However, surgery may not be advisable for patients with higher ASA scores due to the increased perioperative risks. The volumetric limitations are dictated by the size of the tumor itself. The ablation volume was simulated using the Inomed planning software. A maximum of two trajectories were used, with multiple ablations performed along these trajectories to optimize the ablation volume. The maximum ablation diameter achievable with two 10 mm-tip catheters placed in parallel is 44 mm. Consequently, lesions exceeding this maximum diameter at more than one area are too large for a 90% ablation volume. The decision to use only two catheters is primarily practical, to not exceed a critical lesion volume, operating time and MRI use. The “brainstem” location was excluded from our analysis. One of the main complications of LITT is the development of post-therapeutic edema, which has been frequently reported in supratentorial LITT procedures [16, 18]. Data on LITT in the posterior fossa are currently lacking sufficient evidence, but studies point towards a higher morbidity and mortality following LITT in the brainstem [19]. Therefore, we assume, in the absence of contrary evidence, that post-LITT edema may similarly occur in brainstem procedures. Due to the eloquence of the brainstem, which controls vital functions, and the relative proximity of cranial nerve nuclei and fiber tracts, post-procedural edema would be associated with a high risk of potentially life-threatening neurological deterioration. As a result, until proven otherwise, we do not perform LITT in the brainstem at our center. Consequently, we have excluded the brainstem location from this study.

### LITT applicability in our cohort

In our cohort only 17.4% of the analyzed glioma were suitable for LITT. Most cases (82.6%) were not suitable for LITT. Main limiting factors were a multifocal appearance as well as unsuitable shapes and sizes. Other factors (KPS, ASA score, MRI-compatibility) were limiting only in a few cases. In the LITTable cases, simulation showed a wide variety of the complexity regarding the conduction of the procedure. Nearly half of the patients could be treated with only one catheter. Furthermore, the number of ablation points along the catheters were one or two in 41.7% of the cases. The remaining cases would have required an additional catheter or more than two ablations along the catheters. Compared to doing only a stereotactic biopsy, a LITT procedure requires a higher effort. However, the effort to add LITT-ablation to a frame based stereotactic biopsy is comparatively low and recent data showed a similar safety profile for the combined procedure [11].

### Current LITT-applications

Especially for patients with recurrent glioblastoma, which represents a group of patients with limited therapeutic options and poor prognosis, LITT is considered a promising alternative. In this group of patients, the adverse events appeared to be low, while the PFS and OS improved, especially, when adjuvant chemotherapy was applied [12].

Even in pediatric populations the use of LITT for treatment of deep seated and non-safe resectable tumors is emerging. In a recent review, the safety profile was considered relatively low in patients where an open resection was considered too risky [20]. Even in more challenging areas like the cerebellum an application of LITT is reported in pediatric patients [21]. Indeed, the application of LITT might also possible in the posterior fossa with an acceptable safety, as recently demonstrated [22].

The optimal timing of the biopsy and the LITT procedure—whether performed as a combined single-stage intervention or as two separate surgeries—remains a subject of ongoing debate. Recent studies have demonstrated that combining both procedures in a single surgical session does not increase the risk of complications [11]. However, this approach is only feasible when there is sufficient diagnostic certainty, such as in cases of recurrent tumors or where imaging findings clearly indicate a specific diagnosis. In situations where the diagnosis is highly uncertain, a two-step surgical approach is recommended to confirm the diagnosis of glioma before proceeding with LITT. In our series, glioma was already suspected preoperatively in 35 cases, while only one case initially presumed to be lymphoma was later diagnosed as glioma.

### Limiting factors and potential to improve

Recent studies showed, that the extent of ablation is a crucial factor for the outcome after the LITT procedure with the most beneficial effect for near total ablation [23]. To determine a cut off value for the minimal necessary ablation volume for beneficial effects on the outcome is difficult due to the relatively low number of studies and often heterogeneous cohorts. An ablation volume of over 90% is known to be associated with beneficial effects on the outcome [15, 24–26]. However, other studies included patients with a minimal ablation volume of 70%, reporting favorable effects on the outcome at this cut off ablation volume [14, 16]. When an open craniotomy for glioma resection is performed, it is known, that the extent of resection should be at least 90%, which is associated with a better OS and PFS [8]. However, recent studies showed, that even an extent of resection of over 90% is inferior compared to a supramaximal resection regarding the OS and PFS [9]. We think, that the goal of ablation should be comparable to the goal of open surgery with as much ablation as possible but at least 90%. Furthermore, recent studies showed, that the volume of the lesion itself is a factor affecting the outcome after LITT. Smaller lesions (volume under 3 cc) are associated with a better outcome compared to bigger lesions [13]. For bigger lesions as well as lesions with irregular shapes, the use of two trajectories often offers the possibility to achieve a pleasant extent of ablation. Especially in butterfly glioma in the C. Callosum this technique is common [27]. However, the use of two trajectories prolongs the procedure itself and affects the cost effectiveness. Obviously, there are also limiting factors that cannot be influenced through the technique itself. This includes the presurgical ASA-Score, the KPS as well as MRI feasibility. For severe obese patients, an open MRI might enable the MRI feasibility, despite this being the only limiting factor is only of subordinate relevance in our study. Shao et al. reported in a large cohort with over 160 glioma patients a decrease in surgical complications, a lesser amount of trajectories per case as well as an improved outcome with growing experience with the procedure [28]. Therefore, experience as well as a clinical routine are factors that will also positively influence the application of LITT in the future.

### Limitations of this study

This study is limited by its retrospective character. Therefore, our results are based on simulations without actually performing the procedures. Furthermore, it is therefore not known, how many of the patients suitable for LITT would have given informed consent to the procedure. Nevertheless, it allows an estimation of the number of potential candidates for LITT in a real-world cohort of real glioma patients. Beneath that, due to our stereotactic database as the data resource for this study, only glioma patients that received stereotactic biopsies were included. This means, that patients treated with open craniotomy, open biopsy or without surgical treatment at all are not included. Even though the number of patients in the latter group should be relatively low, one could think that some of the patients receiving open craniotomy would consider LITT as an alternative, if they would match the inclusion criteria. However, most of the currently conducted studies regarding LITT in glioma patients are only considering patients not suitable for resection via open craniotomy. Further, patients who declined a stereotactic biopsy are lost and could therefore not be considered. The simulations for this study were conducted exclusively for the Visualase™ system (Medtronic, Minneapolis, MN, USA). This is due to the fact that it is currently the only LITT system approved for use in Europe. Whether a larger number of patients could potentially have been treated using other LITT systems (e.g., Monteris NeuroBlate^®^ or ClearPoint Prism^®^, which are FDA-approved in the USA) were not investigated, as these systems lack approval and accessibility in Europe.

### Outlook

In the future, further studies regarding the effect of LITT in different types of glioma are necessary. Recent studies showed an acceptable safety profile of LITT, enabling prospective controlled trials [16]. Furthermore, data regarding cost-effectiveness of the LITT procedure are currently poor investigated, but the few available studies showed LITT to be favorable compared to craniotomy [29]. Trials aiming to compare the cost effectiveness of the LITT procedure combined with a stereotactic biopsy versus biopsy alone will hopefully give more insights in the cost effectiveness of the LITT procedure [30]. Future studies should investigate on the applicability of LITT in bigger real-world cohorts and, if prospective studies will suggest a comparable safety and outcome profile of the LITT procedure compared to open craniotomy for primary resectable glioma, LITT could also be offered to suitable patients in these group of patients. Further studies should also explore the LITT associated disruption of the blood-brain barrier (BBB) as a potentially beneficial side effect of LITT. Several studies have demonstrated that LITT can disrupt the BBB in vivo [31–33]. This disruption enhances the delivery of chemotherapeutic agents to brain tissue. Currently, research into this beneficial effect is ongoing, with preliminary findings suggesting enhanced drug delivery and efficacy [34, 35]. This could represent an additional consideration in clinical decision-making when choosing between traditional open surgery and LITT. However, to our knowledge, data comparing outcomes following LITT with a smaller ablation volume and an early start of adjuvant chemotherapy to maximize BBB disruption, versus greater tissue resection via open craniotomy and therefore delayed chemotherapy, are not yet available.

## Conclusion

Even though offering many advantages compared to open resection of glioma LITT is currently suitable only for a circumscribed subset of patients. The applicability could be improved by offering solutions for tumors with higher volumes or irregular shapes. Further studies are needed to compare the long-term outcomes for glioma patients treated by LITT with conventionally treated patients. The next goal in LITT research should be to define a group of glioma patients, that benefits the most of the LITT procedure. If these limitations and gap of knowledge can be overstepped, LITT could improve the outcome of more glioma patients in the next years.

## Data Availability

The datasets generated and analyzed during the current study are available from the corresponding author on reasonable request.

## Abbreviations

ASA: American society of anesthesiologists
CT: computer tomography
EOA: Extend of ablation
EOR: Extent of resection
IDH: Isocitrate dehydrogenase
KPS: Karnofsky performance status scale
LITT: Laser interstitial thermal therapy
mm: Millimeter
MGMT: O6-methylguanine-DNA methyltransferase
MRI: Magnet resonance imaging
NIHSS: National institutes of health stroke scale
OS: Overall survival
PACS: Picture archiving and communication system
PFS: Progression free survival
WHO: World health organization
